# Objectification of Skin Firmness: In Vivo Evaluation of 300 Women in Relation to Age

**DOI:** 10.1111/jocd.16773

**Published:** 2025-01-08

**Authors:** Alena Roessle, Martina Kerscher

**Affiliations:** ^1^ Cosmetic Science, Institute of Biochemistry and Molecular Biology University of Hamburg Hamburg Germany

**Keywords:** Cutometer—Corneometer, emergent perceptual categories objectification, reference ranges, skin aging—skin firmness, skin quality

## Abstract

**Background:**

The concept of “skin quality” (SQ) has gained widespread attention, with a recent international consensus defining it and outlining four “emergent perceptual categories” (EPCs), each accompanied by specific parameters and associated measurement methods. No research has confirmed whether the parameters linked to these EPCs vary objectively with age. This gap in data is significant, as understanding how these parameters correlate with age could be essential for creating an objective, age‐adjusted classification of SQ.

**Aim:**

The aim of this study was to investigate the EPC skin firmness in female facial and non‐facial skin in relation to age using biophysical measurements. Reference ranges for objective assessment were determined.

**Patients/Methods:**

Three hundred healthy women (20–69 years) were divided into five age groups. The correlation between age and skin firmness measured by Cutometer and Corneometer (Courage+Khazaka electronic GmbH, Cologne, Germany) devices was evaluated across five anatomical sites: forehead, cheek, neck, décolleté, and the hand. Percentiles were used to generate reference ranges.

**Results:**

Statistical analysis discloses that R2 (*U*_a_/*U*_f_), R5 (*U*_r_/*U*_e_), and R7 (*U*_r_/*U*_f_) correlated with age for all five sites and are preferably assigned to the EPC skin firmness, whereby R2 and R7 showed the strongest correlation. For the neck, significant age‐related changes were found in most of the Cutometer parameters. The stratum corneum (SC) hydration showed only low correlations with age.

**Conclusions:**

R2, R5, and R7 are reliable indicators of age‐related changes in skin firmness, with established reference ranges that can aid in treatment decisions and SQ assessments.

## 1 Introduction

The skin, the body's largest organ, serves as a protective barrier against external elements. Its structure consists of three main layers, with key components in the dermis, such as collagen and elastin, enabling the skin to stretch and then return to its original form [1]. The hydration of the outermost layer, known as the stratum corneum (SC), also plays a role in maintaining the skin's biomechanical properties [2]. Various internal and external factors drive the skin aging process, notably a reduction in collagen production due to decreased fibroblast activity. This decline leads to changes in skin structure, including a reduction in the thickness and resilience of the skin. Additionally, essential elements of the extracellular matrix, like glycosaminoglycans and proteoglycans—highly effective at binding water—begin to diminish with age. These changes contribute to visible signs of aging, such as fine lines, wrinkles, and sagging [3, 4]. The combined loss of mechanical stability and elasticity is a primary marker of skin aging and a critical aspect of overall skin quality (SQ).

An international advisory board of 10 dermatologists and aesthetic physicians defined good SQ as healthy, youthful, and undamaged skin. In this context, youthfulness refers to the perceived age of the skin in relation to the individual's actual biological age. Additionally, the board defined four “emergent perceptual categories” (EPCs) that describe SQ across different ethnicities, age groups (AGs), and genders: skin firmness, skin surface evenness, skin tone evenness, and skin glow. Specific parameters and measurement methods were recommended to assess these EPCs objectively. For instance, skin firmness—encompassing elasticity, tautness/ tightness, and hydration—can be quantified using instruments such as the Cutometer and Corneometer (Courage+Khazaka electronic GmbH, Cologne, Germany) [5]. The significance of skin firmness as a marker of SQ is supported by research from Eiben‐Nielson and Kerscher, who validated the Scientific Assessment Scale of Skin Quality (SASSQ). This scale highlights six parameters, with elasticity and wrinkles showing the highest correlation with age [6]. Through such methods, EPC parameters provide a structured approach to assessing SQ.

To explore whether SQ can be objectively measured in relation to age using the parameters outlined for skin firmness in the EPCs, the first step involves correlating these parameters with age to establish reference ranges. Currently, no data have been published on the objective changes in EPC markers with age, which remains an essential area of study to advance age‐specific classification of SQ.

## 2 Materials and Methods

The study was conducted in accordance with the guidelines of the Declaration of Helsinki and the International Conference of Harmonization Guidelines for Good Clinical Practice. The Independent Ethics Committee—Ethikkommission der Ärztekammer Hamburg—approved the study (2022‐100 840‐BO‐ff).

Three hundred Caucasian females aged between 20 and 69 years were included in this study having given written informed consent. The inclusion and exclusion criteria are listed in Table 1. Participants were divided into cohorts of 60 across five AGs: I: 20–29 years, II: 30–39 years, III: 40–49 years, IV: 50–59 years, and V: 60–69 years.

**TABLE 1:** Inclusion criteria for study participation.

Inclusion criteria	Exclusion criteria
Fitzpatrick skin type (FST) I–IV	BMI < 18.5 or ≥ 30
20‐ to 49‐year‐old prerequisites	Anything that could interfere with the measurement site (e.g., scars and tattoos)
• Premenopausal	
• Follicular phase of the menstrual cycle	
• No change in hormonal contraception within the last 3 months	
50^+^‐year‐old prerequisites	Previous/current tanorexia or severe sunburns
• Postmenopausal	
• Without hormone replacement therapy (HRT) for at least 1 year	
Unchanged skin care routine for at least 3 months	Skin aging signs beyond the normal age range
Healthy skin and a stable state of health	Pregnancy/breastfeeding
	Excessive nicotine consumption (≥ 20 cigarettes/week for more than 2 years)
	Alcohol and drug abuse
	Any disease and treatment that could affect the appearance of the skin
	• Phototherapy or chemotherapy in the last 6 months
	• Prescription oral or topical anti‐wrinkle or skin improvement products within the last 3 months
	• Immunosuppressive and/or immunomodulatory drugs or systemic corticosteroids
	• Cosmetic/medical treatments within the last 3 months
	• Any (minimally) invasive treatment in the measurement sites
	Smoking and/or drinking caffeine prior/during the visit
	Topical applications within the last 12 h
	Contact with water within the last 6 h
	Exposure to extreme cold (e.g., cryotherapy) or heat (e.g., sauna)
	Exercised and/or consumed alcoholic beverages within the last 24 h
	Participation in another clinical study within the last 30 days

**FIGURE 1:**
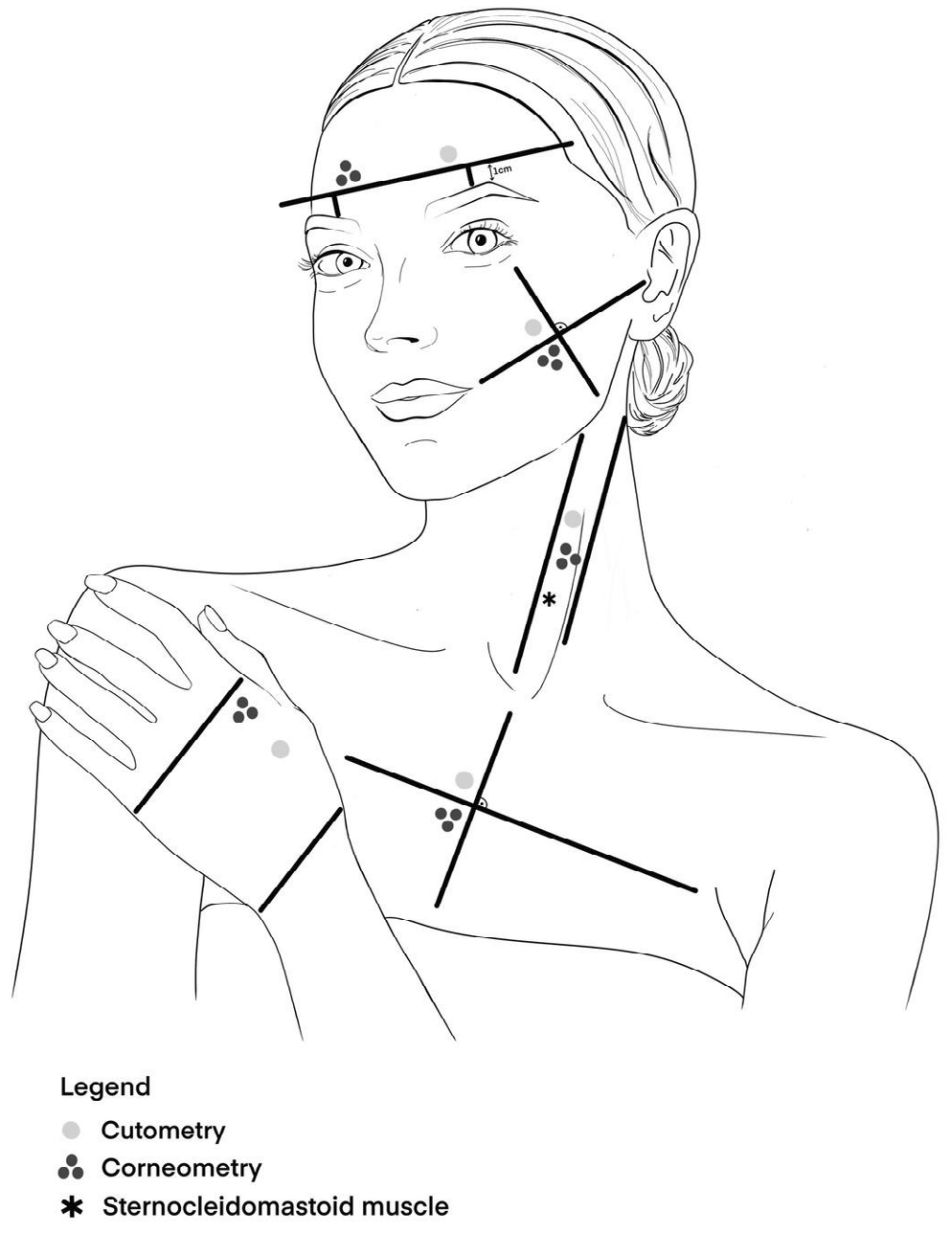
Measurement sites.

Measurements were taken from five skin areas—forehead, cheek, neck, décolleté, and dorsum of the hand—with the right or left side randomly assigned (Figure 1). All measurements were performed by the same investigator following a 30‐min acclimatization period, and adhering to the recommendations of the European Group for Efficacy Measurements on Cosmetics and Other Topical Products were applied (20°C–22°C and 40%–60% humidity) [7, 8].

The Corneometer CM 825 (Courage+Khazaka electronic GmbH, Cologne, Germany) was used to measure the hydration level of the SC at a depth of approximately 10–20 μm. This technique is based on the capacitance measurement of a dielectric medium, which provides an indication of moisture content. For each skin area, three measurements were performed on adjacent sites. Hydration was recorded on a scale from 0 to 130 CM units, with specific thresholds: values below 30 CM units indicated very dry skin, values between 30 and 40 CM units indicated dry skin, and values above 40 CM units indicated well‐hydrated skin [9, 10].

The Cutometer MPA 580 (Courage+Khazaka electronic GmbH, Cologne, Germany) was utilized to assess the biomechanical properties of the skin through a non‐invasive suction‐based method as described previously [8, 11]. The procedure was conducted at a single measurement site, using a 2‐mm probe that applied a negative pressure of 450 mbar. Each cycle consisted of 2 s of suction followed by 2 s of relaxation, repeated five times. Measurements were recorded as absolute values in millimeters, and relative parameters were presented as a ratio. The specific R‐parameters and their significance are detailed in Table 2.

**TABLE 2:** Overview of the Cutometer parameters adapted from Dobrev [11].

R‐parameters [unit]	International *U*‐parameters	Description
R0 [mm]	*U*_f_ (*U*_e_ + *U*_v_)	Skin firmness: first max. amplitude of the suction phase
R1 [mm]	*U*_f_—*U*_a_	Residual deformation/recoverability: difference of first max. and first min. amplitude after one measuring cycle
R2 [%]	*U*_a_/*U*_f_	Gross elasticity/overall visco‐elasticity: ratio of total retraction (min. amplitude) to total distension (max. amplitude)
R3 [mm]	*U*_fx_ ^a^	Distension after repeated cycles: last max. amplitude at the end of the suction phase
R4 [mm]	*U*_ax_ ^a^	Maximum recovery: last min. amplitude after repeated deformations
R5 [%]	*U*_r_/*U*_e_	Net elasticity (without viscous part): ratio of immediate retraction to immediate distension
R6 [%]	*U*_v_/*U*_e_	Ratio of delayed (viscoelastic) to immediate (elastic) distension
R7 [%]	*U*_r_/*U*_f_	Ratio of immediate (elastic) retraction to first max. amplitude
R8 [mm]	*U*_a_	Total recovery: first min. amplitude
R9 (R3 – R0) [mm]	*U*_fx_ ^a^ – *U*_f_	Skin tiring: difference of last max. amplitude and first max. amplitude

*Note: U*_e_: immediate distension—first (elastic) part of the deformation during the suction phase; *U*_v_: delayed distension—second (viscoelastic) part of the deformation during the suction phase; *U*_r_: immediate recovery—first (elastic) part of the retraction during the relaxation phase; *U*_d_ (*U*_a_ – *U*_r_): delayed recovery—second (viscoelastic) part of the retraction during the relaxation phase.

^a^ *x* = 5.

All statistical analyses were performed using IBM SPSS Version 26 (IBM Corp., Armonk, NY, USA). The mean average of the three Corneometer measurements taken at adjacent skin sites was calculated and used in further evaluation.

To examine the linear relationship between age and each parameter, the Bravais–Pearson correlation was applied, with effect sizes categorized according to Cohen's definitions: low (*r* ≥ 0.1), moderate (*r* ≥ 0.3), and high (*r* ≥ 0.5). A significance level of 5% (*p* ≤ 0.05) was used. Only parameters showing a moderate to high Pearson correlation with age were selected for developing reference ranges. These ranges were created to classify measured values as below average, average, or above average.

To define the reference ranges, diagrams were generated based on the 25th and 75th percentiles, with the interquartile range (IQR) illustrating the central tendency and variability within AGs. This approach helps identify and categorize any deviations in measured values clearly.

All assumptions for the statistical tests were reviewed and met. Mild and extreme outliers were retained as true outliers in the dataset after verifying the raw data. The results are presented as mean values with their standard deviations (SDs) (Table S1).

## 3 Results

Data were collected from 300 patients under standardized conditions (temperature 20.35°C ± 1.52°C, relative humidity 51.85% ± 6%) from September 2022 to March 2024. The distribution of demographic data is provided in Table 3.

**TABLE 3:** Demographic data of the assigned age groups.

Groups	Range	Age (Mean ± SD)	*n* (*N* = 300)	Fitzpatrick skin type
AG I	20–29 years	25.1 ± 2.8	60	I: 10; II: 35; III: 11; IV: 4
AG II	30–39 years	32.5 ± 2.4	60	I: 14; II: 33; III: 11; IV: 2
AG III	40–49 years	44.3 ± 2.8	60	I: 13; II: 26; III: 15; IV: 6
AG IV	50–59 years	54.9 ± 2.6	60	I: 10; II: 26; III: 20; IV: 4
AG V	60–69 years	63.5 ± 3.0	60	I: 18; II: 26; III: 16

**FIGURE 2:**
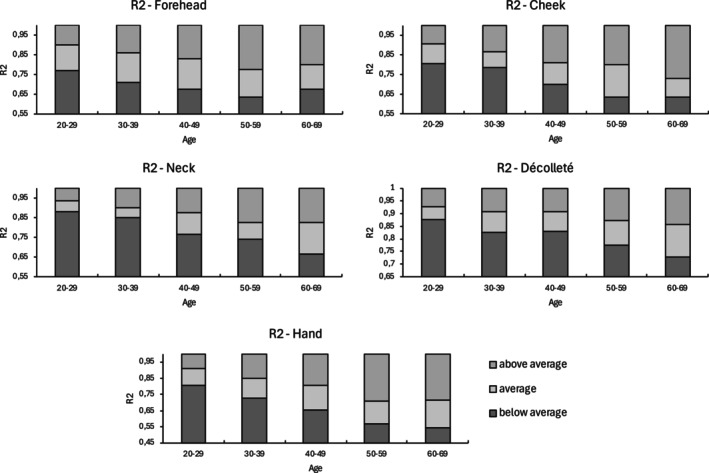
Reference values of R2 for five anatomical sites.

**FIGURE 3:**
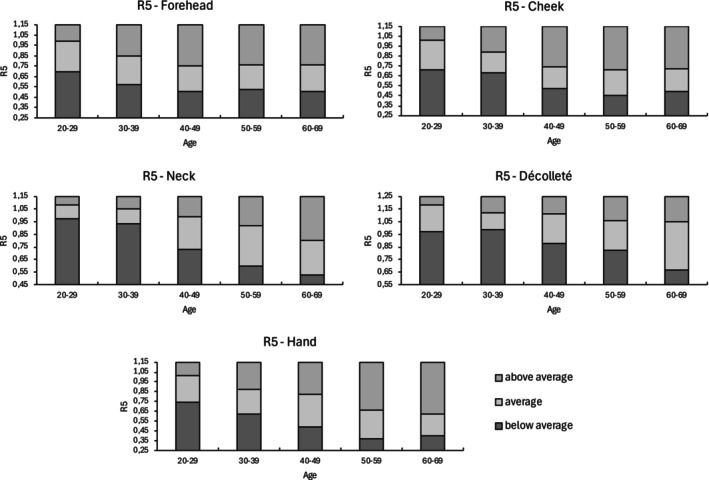
Reference values of R5 for five anatomical sites.

**FIGURE 4:**
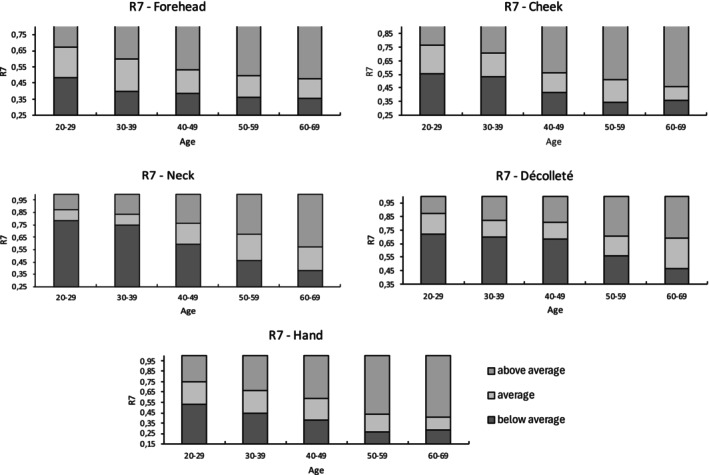
Reference ranges of R7 for five anatomical sites.

Table 4 presents the correlation coefficients of each parameter with age across different measurement sites. Using the IQRs, reference ranges were established for parameters demonstrating the strongest age‐related correlations (R2, R5, and R7—Figure S1). These reference ranges are displayed graphically along with a classification system that categorizes values as below average, average, or above average (Figures 2, 3, 4).

**TABLE 4:** Correlation coefficients between age and the parameters of each site.

Variable	Forehead	Cheek	Neck	Décolleté	Hand
SC hydration	−0.039	−0.127*	0.079	−0.267***	−0.155**
R0	−0.119*	−0.148*	−0.167**	−0.101	−0.128*
R1	0.160**	0.384***	0.450***	0.275***	0.403***
R2	−0.357***	−0.585***	−0.613***	−0.425***	−0.603***
R3	−0.104	−0.131*	−0.120*	−0.077	−0.115*
R4	0.142*	0.333***	0.525***	0.303***	0.412***
R5	−0.319***	−0.472***	−0.546***	−0.386***	−0.561***
R6	0.200**	0.365***	0.417***	0.359***	0.280***
R7	−0.456***	−0.601***	−0.691***	−0.544***	−0.645***
R8	−0.183**	−0.269***	−0.310***	−0.163**	−0.282***
R9	0.058	0.071	0.205***	0.031	−0.057

* *p* < 0.05.

** *p* < 0.01.

*** *p* < 0.001.

### 3.1 Forehead

Parameters R2 (*r* = −0.357, *p* < 0.001), R5 (*r* = −0.319, *p* < 0.001), and R7 (*r* = −0.456, *p* < 0.001) showed significant moderate negative correlations with age. In contrast, parameters R0 (*r* = −0.119, *p* < 0.05), R1 (*r* = 0.160, *p* < 0.01), R3 (*r* = −0.104, *p* > 0.05), R4 (*r* = 0.142, *p* < 0.05), R6 (*r* = 0.200, *p* < 0.01), R8 (*r* = −0.183, *p* < 0.01), and R9 (*r* = 0.058, *p* > 0.05) showed correlations with increasing age close to 0. Additionally, SC hydration (*r* = −0.039, *p* > 0.05) did not correlate with age.

### 3.2 Cheek

The parameters R2 (*r* = −0.585, *p* < 0.001) and R7 (*r* = −0.601, *p* < 0.001) exhibited a significantly high negative correlation with age. Additionally, significant moderate positive correlations with age were observed for R1 (*r* = 0.384, *p* < 0.001), R4 (*r* = 0.333, *p* < 0.001), and R6 (*r* = 0.365; *p* < 0.001), while R5 (*r* = −0.472; *p* < 0.001) displayed a significant moderate negative correlation with age. Parameters R0 (*r* = −0.148, *p* > 0.05), R3 (*r* = −0.131, *p* < 0.05), R8 (*r* = −0.269, *p* < 0.001), and R9 (*r* = 0.071, *p* > 0.05) showed correlations with age close to 0. Similarly, SC hydration (*r* = −0.127, *p* < 0.05) did not correlate with age.

### 3.3 Neck

Parameters R2 (*r* = −0.613, *p* < 0.001), R5 (*r* = −0.546, *p* < 0.001), and R7 (*r* = −0.691, *p* < 0.001) showed significantly high negative correlations with age, while R4 (*r* = 0.525, *p* < 0.001) demonstrated a significantly high positive correlation with age. Significant moderate positive correlations with age were found for Cutometer parameter R1 (*r* = 0.450, *p* < 0.001) and R6 (*r* > 0.417, *p* < 0.001), while R8 (*r* = −0.310, *p* < 0.001) showed a significant moderate negative correlation. Parameters R0 (*r* = −0.167, *p* < 0.01), R3 (*r* = −0.120, *p* < 0.05), and R9 (*r* = 0.205, *p* < 0.001) had correlations with age close to 0. Additionally, SC hydration (*r* = 0.079, *p* > 0.05) also showed no correlation with age.

### 3.4 Décolleté

The parameter R7 (*r* = −0.544, *p* < 0.001) exhibited a significantly high negative correlation with age. A significant moderate positive correlation with age was found for R4 (*r* = 0.303, *p* < 0.001) and R6 (*r* > 0.359, *p* < 0.001), and significant moderate negative correlations for R2 (*r* = −0.425, *p* < 0.001) and R5 (*r* > −0.386, *p* < 0.001). The SC hydration showed a low effect size with an *r* of −0.267 (*p* < 0.001). Parameter R0 (*r* = −0.101, *p* > 0.05), R1 (*r* = 0.275, *p* < 0.001), R3 (*r* = −0.077, *p* > 0.05), R8 (*r* = −0.163, *p* < 0.01), and R9 (*r* = 0.031, *p* > 0.05) demonstrated correlations with age near 0.

### 3.5 Hand

The parameters R2 (*r* = −0.603, *p* < 0.001), R5 (*r* = −0.561, *p* < 0.001), and R7 (*r* = −0.645, *p* < 0.001) showed significant high negative correlation with age. Significant moderate positive correlations with age were found for R1 (*r* = 0.403, *p* < 0.001) and R4 (*r* = 0.412, *p* < 0.001). Correlations with age near 0 were found for the parameters R0 (*r* = −0.128, *p* < 0.05), R3 (*r* = −0.115, *p* < 0.05), R6 (*r* = 0.280, *p* < 0.001), R8 (*r* = −0.282, *p* < 0.001), and R9 (*r* = −0.057, *p* > 0.05). SC hydration (*r* = −0.155, *p* < 0.01) also showed no correlation with age.

## 4 Discussion

Even though the four different EPCs are well‐established criteria in aesthetics to improve patients' individual SQ, no objective criteria have been published so far to exactly quantify the individual dimension of each EPC related to the individual's age.

Cutometer and Corneometer are widely used tools for objectively assessing skin condition. As biophysical measurement methods, they were utilized in this study to investigate and objectively measure the SQ EPC of skin firmness across different anatomical sites, including the face (forehead, cheek), neck, décolleté, and hand, in relation to age. Using a 2‐mm Cutometer probe, the measurements specifically reflect the mechanical properties of the epidermis and upper dermis [11].

Based on the analysis of 300 females, the Cutometer parameters R2, R5, and R7 demonstrated significant moderate to high negative correlations with age (*r* = −0.319 to −0.619) across all five measurement sites. Notably, R2 and R7 showed the highest negative correlation coefficients in at least three sites, suggesting that the skin's ability to recover from deformation declines with age. In contrast, parameters R0, R3, and R9 showed the lowest correlation coefficients across all sites.

Our findings align with the results of previous studies, despite variations in methodology, measurement sites, and participation prerequisites (such as ethnicity) [12, 13, 14, 15]. For example, Krueger et al. investigated the mechanical properties of the skin in 120 female subjects across five body areas (cheek, neck, cleavage, forearm, and back of the hand), reporting significant high average correlations for R2, R5, R7 as well as Ur and R8 [12].

Korean studies by Ahn et al. (*N* = 44 women; 20–61 years) and Ryu et al. (*N* = 96 women; 20–75 years), focusing on women's cheeks, also found significant strong negative correlations for these parameters [13, 14]. Ryu et al. further demonstrated a significant positive correlation with age for the parameter R6 [13], indicating a higher proportion of viscoelasticity in the elastic part of the suction phase as age progresses [11]. Consistent with their results, we observed moderate correlations for R6 with age on the cheek, neck, and décolleté. Due to the significant strong correlations of R2 and R7 with age, Krueger et al., Ahn et al., and Ryu et al. highlighted these parameters as important indicators for measuring age‐related changes in skin [12, 13, 14]. However, Ryu et al. found no significant correlation between age and parameters R0, R3, and R9 for the cheek [13], consistent with our findings of low correlations for these parameters at all five sites.

Regarding skin firmness measured by the Cutometer parameter R0, we observed negative correlations with age, consistent with previous studies [12, 13, 15]. Decreasing R0 values align with findings by Luebberding et al., who observed significant negative correlations for R0, R2, R5, R7, R8, *U*_r_, and *U*_e_, across various sites (cheek, neck, volar forearm, and dorsum of the hand) in a cohort of 300 subjects (150 female/150 male; ages 20–74) exploring gender‐related differences in skin's mechanical properties in relation to age. They observed that R0 values decreased with age across all sites, with comparable values for the cheek (AG I = 95.25 ± 34.26 μm; AG IV: 45.26 ± 18.32 μm), neck (AG I = 214.66 ± 72.05 μm; AG IV: 105.30 ± 53.14 μm), and the hand (AG I = 108.16 ± 46.58 μm; AG IV: 53.57 ± 33.06 μm) [15]. In our study, the R0 values for these sites are generally higher, likely due to strict adherence to the inclusion and exclusion criteria, with particular emphasis on healthy skin.

Krueger et al. also reported a significant negative correlation between age and R0 across different sites [12]. In younger subjects, lower R0 values are associated with firmer skin, while in aged skin, the lower R0 values may result from a reduction in the elastic component of the deformation phase (*U*_e_) and an increase in the viscoelastic component (*U*_v_) [11]. When considering potential dermal influences, the proteolytic degradation of collagen is inhibited by mineral deposits and increased advanced glycation end—dependent cross‐linking. This slows the proteolytic turnover rate with age, preventing the degradation of older collagen fibers. Consequently, modified collagen fibers lose functionality and exhibit increased stiffness compared to native fibers [16, 17]. Supporting this, He et al. observed that naturally aged hip skin becomes mechanically firmer due to biological changes in collagen [18].

As R0 and R8 values decrease with age, R1 correspondingly increases, indicating diminished capacity for skin recovery after deformation [11]. This trend is also evident after repeated cycles (five cycles of 2 s suction, 2 s relaxation), where R4 values increase—except on the forehead which shows only a low correlation coefficient. The forehead exhibits the least correlation with Cutometer parameters, suggesting that additional factors, such as the exposome, may have a greater influence on this region than biological aging.

If the definitions of elasticity and tautness/tightness are adopted from Goldie et al. for assessing skin firmness using Cutometer parameters R0 and R2 [5], R0 appears less suitable for age‐related studies. In contrast, R2 is recommended as an age‐sensitive parameter, as also indicated by Krueger et al. [12]

Hydration, another component of the skin firmness EPC, according to Goldie et al. [5], was measured with the Corneometer. No significant correlation with age was observed for hydration in the forehead and neck, and only significant low correlations were found for the cheek, décolleté, and hand. Luebberding et al., who examined age‐related changes in skin barrier function (*N* = 150 women; ages 18–80), found stable or increased SC hydration values with age [19]. A 2022 meta‐analysis also identified no significant differences in skin hydration across AGs for the forehead, cheek, and neck. Samadi et al. attributed this to the difference in free versus protein‐bound water content in the skin [20]. The Corneometer may primarily measure free water in the skin, while protein‐bound water—which decreases due to age‐related changes in protein structure—could remain unmeasured, potentially explaining our results [21]. Additionally, SC thickness could play a role; according to Serup et al., higher SC hydration values are associated with a thicker SC [22], yet minimal age‐related differences in SC thickness have been found [23].

It should be noted that data collection for this study occurred over an extended period, which may have influenced the result. However, measurements were predominantly taken in winter, and subjects were not assessed in any specific order.

In conclusion, our study for the first time provides a more detailed objective standardized quantification of EPC skin firmness by using biophysical parameters to investigate age‐related changes in skin structure and composition. The elastic parameters R2, R5, and R7, which demonstrated significant moderate to strong correlations across all anatomical sites (*r* = −0.319 to −0.691), are well‐suited for assessing age‐related changes. These parameters can help guide treatment decisions, establish baseline SQ measures, and monitor improvements through the reference ranges, especially for individuals where skin firmness is a primary concern. For example, Hertz‐Kleptow et al. observed significant improvement in gross elasticity (R2) in the cheek area following injections with CPM‐HA20G (Belotero Revive, Merz Pharmaceuticals GmbH, Frankfurt, Germany), with effects seen from baseline to Weeks 9 and 12 [24]. Kerscher et al. also reported improvements in skin properties with microfocused ultrasound with visualization (MFU‐V; Ultherapy, Merz North America, Raleigh, NC, USA). After a single treatment with MFU‐V, a significant increase in R2 and R5 was measured after 12 and 24 weeks [25]. Such treatments may effectively enhance EPC skin firmness.

SC hydration, on the other hand, showed weaker correlation with age, suggesting that as a superficial parameter, it may be more reflective of skin condition than that of structural aging.

The established reference ranges, from below average to above average, enable an objective classification of EPC skin firmness, providing valuable support for both research and clinical applications. Among the sites assessed, the neck exhibited strong age‐related correlations, followed by the cheek, hand, and décolleté, with the forehead showing the weakest correlations. These findings suggest that the neck should be considered a standard site in clinical trials evaluating mechanical properties.

Future research should be performed to assess changes in skin firmness before and after a suitable treatment regimen to gain a deeper insight into the timeline of changes in SQ.

## Author Contributions

The author, Alena Roessle, is responsible for the conception, design, data collection, data analysis, and writing of this manuscript. Prof. Kerscher provided guidance and advisory support throughout the study process.

## Ethics Statement

The authors confirm that the ethical policies of the journal, as outlined on the journal's author guidelines page, have been adhered to, and appropriate approval from the ethical review committee has been obtained. The Independent Ethics Committee (Ethikkommission der Ärztekammer Hamburg) in Germany approved the study (2022‐100 840‐BO‐ff) on September 20, 2022. The study was conducted in accordance with the principles of the Declaration of Helsinki and the International Conference on Harmonization Guidelines for Good Clinical Practice. Measurements were not taken prior to the signing of the informed consent.

## Conflicts of Interest

The authors declare no conflicts of interest.

## Supporting information


**Data S1.** Supporting Information.
**Table S1**. Mean values ± SD of the age groups per site.
**Figure S1**. Scatter plots of selected Cutometer values per age.

## Data Availability

Upon reasonable request, the data that support the findings of this study are available from the corresponding author.
