# Supplementary material for: Objectification of Skin Firmness: In Vivo Evaluation of 300 Women in Relation to Age
Source: J Cosmet Dermatol. 2025 Jan 8;24(1):e16773. doi: 10.1111/jocd.16773 (PMC11712028; doi:10.1111/jocd.16773)
Supplement: Supplementary file 1 — Data S1. Supporting Information. Table S1. Mean values ± SD of the age groups per site. Figure S1. Scatter plots of selected Cutometer values per age. [file JOCD-24-e16773-s001.docx]

Supplemental Table 1: MEAN Values ± SD OF THE AGE GROUPS PER SITE.

| **Parameter** | **SITES** | | | | |
| --- | --- | --- | --- | --- | --- |
|  | **Forehead** | **Cheek** | **Neck** | **Décolleté** | **Hand** |
| **SC Hydration [CM units]** | | | | | |
| AG I | 54.77 ± 10.49 | 33.19 ± 13.88 | 50.82 ± 10.03 | 52.64 ± 11.73 | 29.71 ± 8.81 |
| AG II | 50.79 ± 10.12 | 33.77 ± 12.02 | 52.90 ± 10.13 | 52.58 ± 11.63 | 27.74 ± 8.24 |
| AG III | 49.75 ± 10.77 | 40.90 ± 11.55 | 53.50 ± 7.71 | 48.08 ± 11.21 | 25.28 ± 8.18 |
| AG IV | 52.75 ± 13.58 | 34.38 ± 13.45 | 55.73 ± 10.64 | 51.85 ± 14.40 | 27.11 ± 8.25 |
| AG V | 52.66 ± 11.33 | 39.62 ± 13.55 | 52.66 ± 11.33 | 39.57 ± 13.52 | 26.30 ± 8.55 |
| **R0 [mm]** | | | | | |
| AG I | 0.217 ± 0.100 | 0.316 ± 0.095 | 0.459 ± 0.119 | 0.285 ± 0.110 | 0.214 ± 0.089 |
| AG II | 0.237 ± 0.112 | 0.352 ± 0.106 | 0.476 ± 0.124 | 0.299 ± 0.103 | 0.224 ± 0.090 |
| AG III | 0.270 ± 0.118 | 0.364 ± 0.107 | 0.520 ± 0.102 | 0.364 ± 0.110 | 0.237 ± 0.085 |
| AG IV | 0.198 ± 0.094 | 0.307 ± 0.108 | 0.445 ± 0.118 | 0.264 ± 0.837 | 0.196 ± 0.072 |
| AG V | 0.188 ± 0.101 | 0.280 ± 0.111 | 0.403 ± 0.108 | 0.262 ± 0.110 | 0.188 ± 0.092 |
| **R1 [mm]** | | | | | |
| AG I | 0.037 ± 0.025 | 0.046 ± 0.025 | 0.048 ± 0.031 | 0.031 ± 0.019 | 0.032 ± 0.020 |
| AG II | 0.045 ± 0.024 | 0.060 ± 0.026 | 0.058 ± 0.025 | 0.037 ± 0.017 | 0.043 ± 0.017 |
| AG III | 0.067 ± 0.053 | 0.090 ± 0.040 | 0.102 ± 0.054 | 0.054 ± 0.037 | 0.070 ± 0.039 |
| AG IV | 0.055 ± 0.030 | 0.091 ± 0.054 | 0.103 ± 0.057 | 0.045 ± 0.022 | 0.073 ± 0.038 |
| AG V | 0.051 ± 0.033 | 0.091 ± 0.050 | 0.110 ± 0.061 | 0.057 ± 0.041 | 0.069 ± 0.046 |
| **R2** | | | | | |
| AG I | 0.831 ± 0.096 | 0.847 ± 0.082 | 0.893 ± 0.074 | 0.890 ± 0.061 | 0.843 ± 0.085 |
| AG II | 0.781 ± 0.112 | 0.818 ± 0.087 | 0.879 ± 0.038 | 0.866 ± 0.062 | 0.788 ± 0.089 |
| AG III | 0.760 ± 0.113 | 0.750 ± 0.084 | 0.814 ± 0.083 | 0.866 ± 0.065 | 0.718 ± 0.116 |
| AG IV | 0.708 ± 0.106 | 0.707 ± 0.102 | 0.774 ± 0.083 | 0.820 ± 0.085 | 0.635 ± 0.112 |
| AG V | 0.723 ± 0.105 | 0.679 ± 0.085 | 0.736 ± 0.099 | 0.783 ± 0.106 | 0.636 ± 0.128 |
| **R3 [mm]** | | | | | |
| AG I | 0.255 ± 0.110 | 0.365 ± 0.101 | 0.510 ± 0.128 | 0.328 ± 0.116 | 0.238 ± 0.092 |
| AG II | 0.278 ± 0.120 | 0.404 ± 0.111 | 0.528 ± 0.123 | 0.346 ± 0.107 | 0.247 ± 0.096 |
| AG III | 0.311 ± 0.122 | 0.416 ± 0.108 | 0.581 ± 0.105 | 0.416 ± 0.118 | 0.266 ± 0.087 |
| AG IV | 0.237 ± 0.099 | 0.360 ± 0.113 | 0.506 ± 0.124 | 0.312 ± 0.091 | 0.221 ± 0.080 |
| AG V | 0.230 ± 0.113 | 0.332 ± 0.120 | 0.466 ± 0.115 | 0.311 ± 0.123 | 0.212 ± 0.101 |
| **R4 [mm]** | | | | | |
| AG I | 0.069 ± 0.053 | 0.094 ± 0.049 | 0.081 ± 0.047 | 0.052 ± 0.033 | 0.045 ± 0.027 |
| AG II | 0.085 ± 0.047 | 0.118 ± 0.043 | 0.097 ± 0.039 | 0.066 ± 0.028 | 0.064 ± 0.027 |
| AG III | 0.116 ± 0.073 | 0.160 ± 0.059 | 0.166 ± 0.073 | 0.087 ± 0.054 | 0.098 ± 0.050 |
| AG IV | 0.094 ± 0.047 | 0.159 ± 0.078 | 0.172 ± 0.084 | 0.079 ± 0.033 | 0.104 ± 0.054 |
| AG V | 0.091 ± 0.060 | 0.152 ± 0.077 | 0.193 ± 0.085 | 0.096 ± 0.062 | 0.099 ± 0.062 |
| **R5** | | | | | |
| AG I | 0.832 ± 0.229 | 0.847 ± 0.234 | 0.992 ± 0.208 | 1.073 ± 0.207 | 0.864 ± 0.209 |
| AG II | 0.736 ± 0.197 | 0.799 ± 0.167 | 1.002 ± 0.124 | 1.048 ± 0.132 | 0.757 ± 0.177 |
| AG III | 0.646 ± 0.173 | 0.650 ± 0.155 | 0.855 ± 0.176 | 1.006 ± 0.181 | 0.628 ± 0.203 |
| AG IV | 0.642 ± 0.177 | 0.601 ± 0.178 | 0.760 ± 0.209 | 0.918 ± 0.191 | 0.541 ± 0.197 |
| AG V | 0.648 ± 0.191 | 0.602 ± 0.165 | 0.688 ± 0.217 | 0.846 ± 0.246 | 0.521 ± 0.191 |
| **R6** | | | | | |
| AG I | 0.460 ± 0.198 | 0.332 ± 0.120 | 0.254 ± 0.108 | 0.364 ± 0.103 | 0.366 ± 0.112 |
| AG II | 0.423 ± 0.143 | 0.317 ± 0.087 | 0.270 ± 0.126 | 0.391 ± 0.119 | 0.354 ± 0.112 |
| AG III | 0.412 ± 0.144 | 0.316 ± 0.099 | 0.267 ± 0.112 | 0.358 ± 0.129 | 0.367 ± 0.128 |
| AG IV | 0.501 ± 0.158 | 0.412 ± 0.124 | 0.347 ± 0.129 | 0.465 ± 0.109 | 0.441 ± 0.135 |
| AG V | 0.539 ± 0.203 | 0.456 ± 0.174 | 0.434 ± 0.163 | 0.497 ± 0.177 | 0.451 ± 0.150 |
| **R7** | | | | | |
| AG I | 0.568 ± 0.146 | 0.633 ± 0.166 | 0.788 ± 0.150 | 0.783 ± 0.124 | 0.631 ± 0.142 |
| AG II | 0.515 ± 0.125 | 0.608 ± 0.123 | 0.791 ± 0.073 | 0.756 ± 0.097 | 0.561 ± 0.125 |
| AG III | 0.452 ± 0.099 | 0.492 ± 0.105 | 0.675 ± 0.119 | 0.739 ± 0.105 | 0.470 ± 0.129 |
| AG IV | 0.425 ± 0.098 | 0.422 ± 0.106 | 0.563 ± 0.143 | 0.627 ± 0.125 | 0.371 ± 0.125 |
| AG V | 0.415 ± 0.081 | 0.409 ± 0.086 | 0.476 ± 0.135 | 0.565 ± 0.153 | 0.357 ± 0.120 |
| **R8 [mm]** | | | | | |
| AG I | 0.183 ± 0.087 | 0.265 ± 0.093 | 0.397 ± 0.118 | 0.269 ± 0.115 | 0.192 ± 0.079 |
| AG II | 0.170 ± 0.099 | 0.280 ± 0.087 | 0.408 ± 0.114 | 0.244 ± 0.090 | 0.158 ± 0.080 |
| AG III | 0.223 ± 0.097 | 0.284 ± 0.104 | 0.433 ± 0.088 | 0.309 ± 0.102 | 0.178 ± 0.073 |
| AG IV | 0.154 ± 0.073 | 0.228 ± 0.076 | 0.358 ± 0.088 | 0.240 ± 0.089 | 0.145 ± 0.060 |
| AG V | 0.132 ± 0.079 | 0.199 ± 0.080 | 0.306 ± 0.080 | 0.213 ± 0.093 | 0.119 ± 0.058 |
| **R9 [mm]** | | | | | |
| AG I | 0.038 ± 0.017 | 0.049 ± 0.014 | 0.051 ± 0.015 | 0.043 ± 0.014 | 0.025 ± 0.015 |
| AG II | 0.041 ± 0.022 | 0.052 ± 0.013 | 0.052 ± 0.015 | 0.047 ± 0.018 | 0.023 ± 0.012 |
| AG III | 0.042 ± 0.019 | 0.056 ± 0.028 | 0.067 ± 0.048 | 0.057 ± 0.040 | 0.026 ± 0.015 |
| AG IV | 0.039 ± 0.013 | 0.053 ± 0.015 | 0.061 ± 0.021 | 0.048 ± 0.015 | 0.025 ± 0.013 |
| AG V | 0.043 ± 0.019 | 0.053 ± 0.014 | 0.067 ± 0.022 | 0.045 ± 0.019 | 0.020 ± 0.012 |


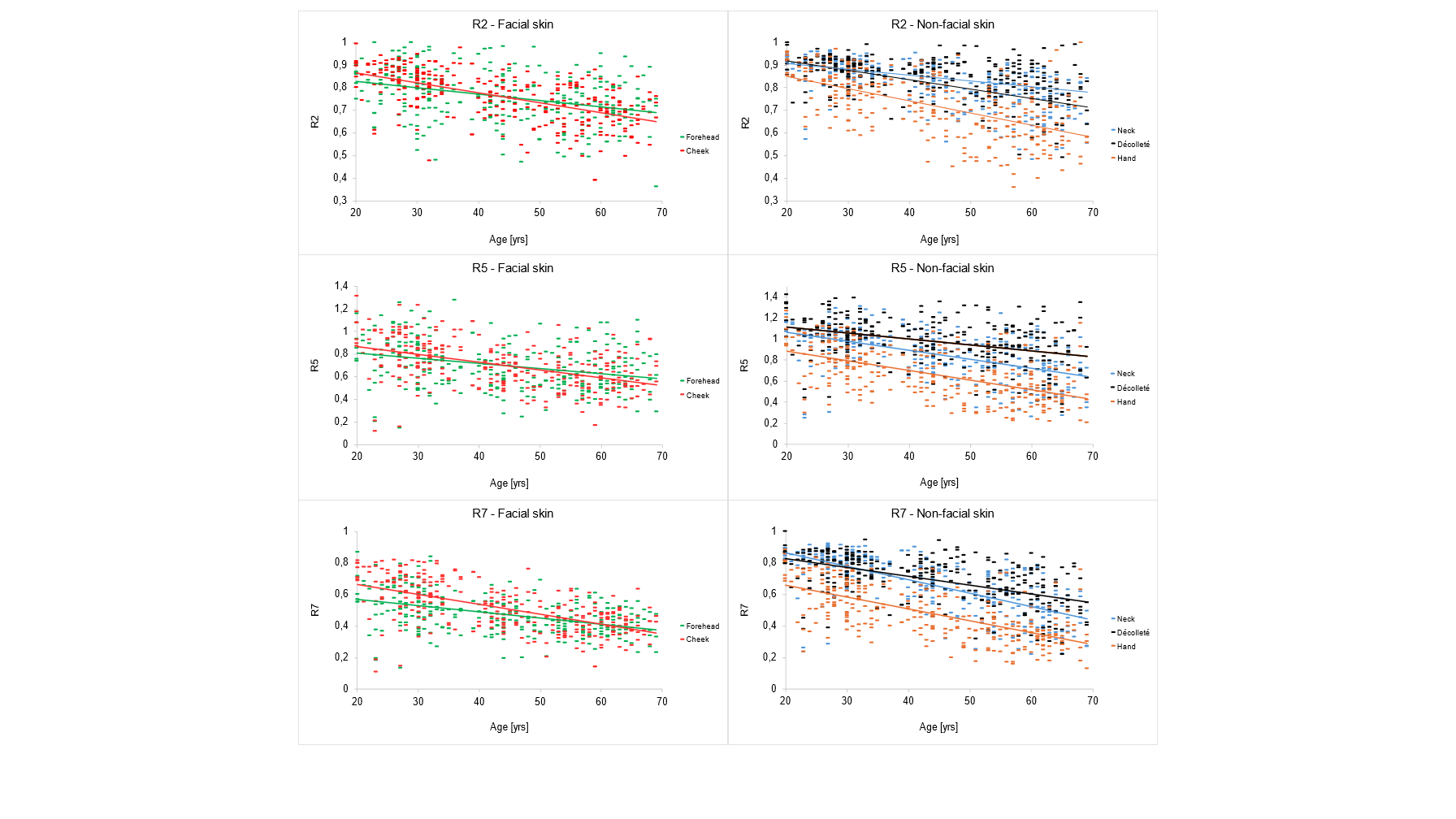


Supplemental Figure 1:SCATTER PLOTS OF SELECTED CUTOMETER^®^ VALUES PER AGE.
